# Aspirin wears smart

**DOI:** 10.1093/ehjcvp/pvx017

**Published:** 2017-05-15

**Authors:** Vincenzo Mollace, Giuseppe Rosano, Natalia Malara, Enzo Di Fabrizio, Cristiana Vitale, Marialaura Coluccio, Jessica Maiuolo, Ayesha Ali Wasti, Carolina Muscoli, Micaela Gliozzi, Rocco Mollace, Vincenzo Musolino, Cristina Carresi, Massimo Fini, Bruno Silvestrini

**Affiliations:** 1 Department of Health Sciences, Institute of Research for Food Safety & Health, Nutramed Scarl, University of Catanzaro “Magna Graecia”, Catanzaro, Italy;; 2 San Raffaele IRCCS Pisana, Rome, Italy;; 3 Department of Experimental and Clinical Medicine, University of Magna Graecia, Catanzaro, 88100, Italy;; 4 King Abdullah University of Science and Technology, Thuwal, 23955-6900, Saudi Arabia;; 5 SBM srl, Italy

Low-dose aspirin is used worldwide for preventing thromboembolic disorders. Its use, however, is often associated with gastrointestinal bleeding, mostly due to direct irritation of the gastric mucosa. Here we provide evidence for a novel sublingual formulation of aspirin micronized and co-grinded with collagen proven to be as effective as oral standard formulation in inhibiting platelet aggregation but with attenuated gastric irritation. This represents a new option for better aspirin treatment in the prevention of myocardial infarction and stroke.

Orally given low-dose aspirin has been used for decades due to its anti-inflammatory and antithrombotic properties.^1^ Although standard oral formulation of aspirin allows rapid and complete absorption from the GI tract, new formulations have been developed and marketed, (e.g. dry granules, effervescent solution, and chewable tablets)^2^^,^^3^ with the aim to achieve faster dissolution and faster absorption^4^^,^^5^ as well as to reduce direct aspirin-induced gastric lesions. However, the occurrence of gastrointestinal bleeding still remains a significant problem of chronic aspirin administration and, sometimes, limits the use of aspirin in primary prevention.^6^^,^^7^ On the other hand, co-administration of proton pump inhibitors is currently used to counteract aspirin-induced gastric lesions,^8^^,^^9^ thereby representing a pharmaco-economic issue in the area of health care sustainability.

Recently, we developed and patented (N. 102015000079955) a new formulation of aspirin which leads to faster absorption and activity but devoid of direct gastrointestinal lesioning effect. In addition, this formulation allows sublingual administration of the drug which, by passing the liver metabolism, leads to faster serum peak concentration with more prominent and rapid inhibition of cycloxygenase (COX), the major target of antiplatelet and antinflammatory action of aspirin.

## Methods

**Figure 1:**
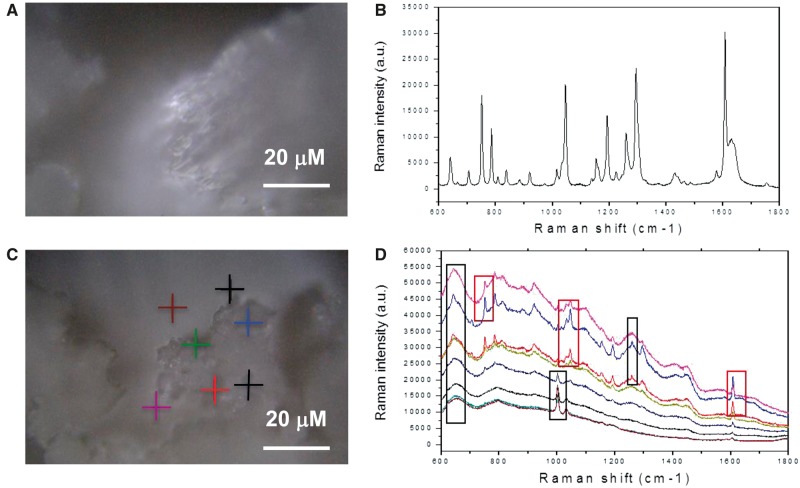
Micro-Raman spectra were excited by a 514 nm laser line through a 50X objective with a laser power of 10 mW at the sample level. The samples were deposited as powder on a calcium fluoride slide, and measurements were performed with an accumulation time of 30 s, in the range from 500 up to 4000 cm^−1^. In (*A*) the image of the chemical compost aspirin in crystalline form. Raman spectra in (*B*) relative to the analysis of the compost. The measures yield a spectrum with very sharp, intense Raman peaks. In (*C*) the optical image of the dried mixture composed of the amorphous material and the crystalline aspirin. The amorphous form is due to the presence of collagen. RAMAN measurements (*D*), collected in the marked points of the optical image, show broader less intense Raman peaks (indicated by the black square) in the presence of the amorphous species and sharp peaks (red squares) related to aspirin molecules.

Cristalline aspirin was amorphized via micronization and co-grinding with collagen (see Supplementary material online, Materials & Methods). Collagen was chosen due to its gastro-protective action.^10^ The occurrence of aspirin amorphization was demonstrated via measurement of spectra collected with Raman spectroscopy (*Figure 1*).

Micronized collagen co-grinded aspirin formulations were developed for both oral and sublingual administration (see Supplementary material online, Materials & Methods) and used in healthy volunteers, in a phase 1, randomized, double blind, placebo-controlled study (study EudraCT N. 2013-002980-24) to verify their pharmacokinetic profile compared to standard crystalline formulations of aspirin (see Supplementary material online, Clinical Study Protocol and healthy volunteer demographics). Measurement of serum thromboxane B_2_ (TXB_2_), and its urinary metabolite 11-dehydro-TXB_2_ after both oral and sublingual standard as well as micronized aspirin were carried out to assess the inhibitory activity at the COX level.

## Results

**Figure 2:**
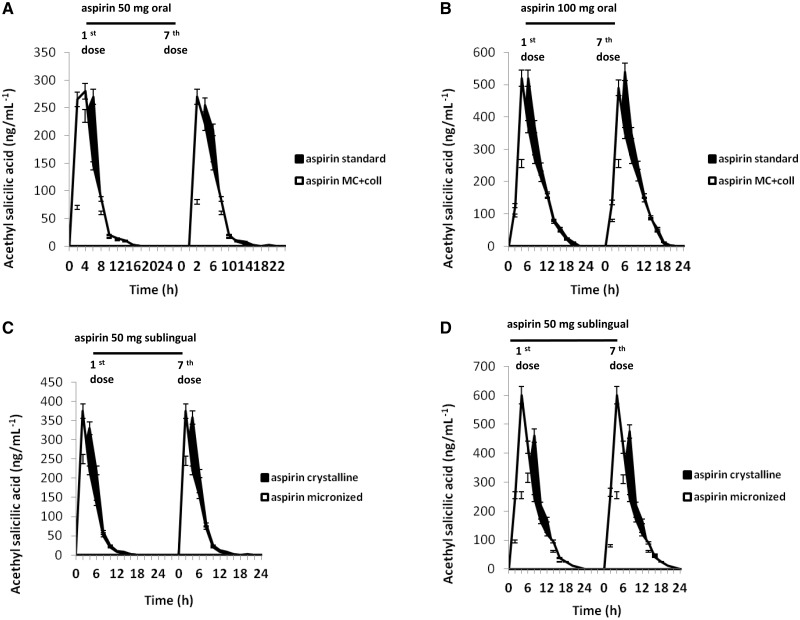

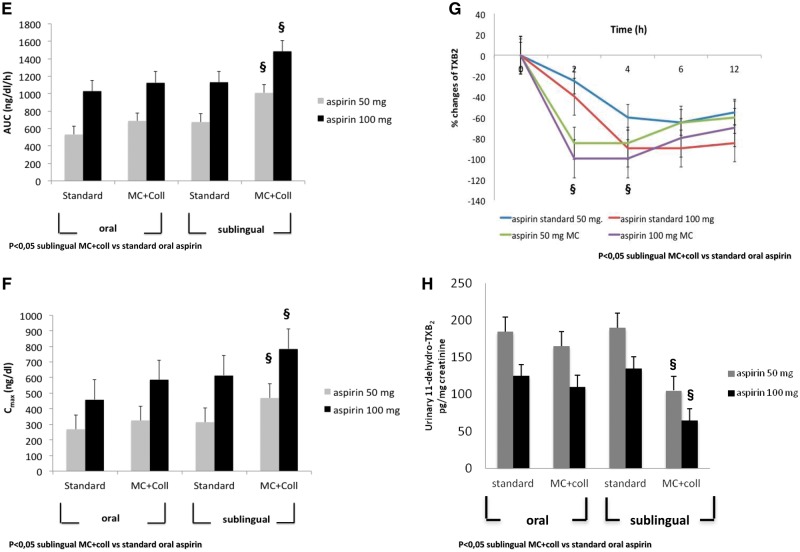
Plasma concentration vs. time profile of acetylsalicylic acid after oral and sublingual administration of aspirin either standard crystalline and micronized and co-grinded with collagen (MC + coll) (detected via LC-MS and expressed as ng/mL^−1^) of healthy subjects (*n* = 10 for each treatment) after dosing with 50 and 100 mg/daily of both formulations (Graphs *A*–*D*). Curves display changes of levels of acetylsalicylic acid after administration of the first and seventh dose of aspirin of both formulations. Graphs *E* and *F* show the area under the curve (AUC calculated as ng/dL/h) and *C*_max_ (expressed as ng/dL), respectively, of acetylsalicylic acid serum concentrations after oral or sublingual administration of both aspirin formulations (standard or MC + coll) given in healthy volunteers (*n* = 10 for each treatment). In *G* are shown the changes of TXB_2_ serum levels detected via ELISA immunoassay in subjects treated with oral standard aspirin vs. sublingual administration of MC + coll formulation. Data show that the decrease of serum TXB_2_ occurs earlier in subjects treated sublingually with aspirin MC + coll compared to the standard oral administration of 50 and 100 mg, respectively. Similar effect was seen in urinary 11-dehydro-TXB_2,_ the metabolite of TXB2, thus reflecting a better effect of sublingual aspirin MC + coll formulation on platelet COX enzyme.

Oral administration of 100 mg of standard crystalline aspirin (*n* = 10 healthy volunteers) for 7 consecutive days produced a rapid rise of acetylsalicylic acid serum concentration which peaked at 2–4 h after the administration and declined 8–12 h later (*Figure 2–F*). This effect was accompanied by a decrease of TXB_2_ serum concentration at 6 h which declined 16–20 h after the administration. (*Figure 2G*). A similar response was seen in healthy volunteers (*n* = 10) receiving 50 mg of standard aspirin orally, with lower effect compared to 100 mg, (*Figure 2A–F*). No significant differences were seen when 50 and 100 mg of micronized and collagen co-grinded aspirin were given orally to healthy volunteers (*n* = 10 for each dose; *Figure 2A–F*)

In contrast, sublingual administration of 50 and 100 mg (*n* = 10 for each dose) of aspirin micronized and co-grinded with collagen, produced a dose-related peak of serum concentration of acetylsalicylic acid which occurred earlier compared to both sublingual and oral administration of aspirin (1 h) with a decline of serum concentration occurring 4–5 h after the administration (*n* = 10 for each dose; *Figure 2A–F*). This effect was accompanied by early inhibition of TXB_2_ levels which was observed after 2 h and lasted 10 h after aspirin administration. Furthermore, the effect of aspirin formulation in TXB_2_ was confirmed by detection of its urinary metabolite11-dehydro-TXB_2_ after Day 7 of the study (*Figure 2H*), thus suggesting that sublingual administration of micronized co-grinded aspirin displays a non-inferiority response on COX enzyme compared to crystalline standard formulation. Determination of serum TXB_2_ serum levels and of urinary 11-dehydro-TXB_2_ showed no changes before and after treatment in healthy volunteers receiving placebo (not shown).

Neither changes on routine blood analytical biomarkers nor side effects or adverse drug reactions were noted in any of the groups after administration of oral or sublingual aspirin. Pill count adherence was 100% and no enrolled subject was excluded from the study (see Supplementary material online).

Finally, to verify the attenuated impact on gastric mucosa of micronized collagen co-grinded aspirin compared to standard oral formulation, experiments have been carried out in rats receiving doses of aspirin proven to produce gastric lesions (see Supplementary material online, Materials & Methods). In particular, acute oral administration of aspirin (400 mg/Kg) both crystalline or micronized and co-grinded with collagen produced gastric lesion with an elevated ulcer score index.^11^ In particular 83% of the stomachs in the group of rats treated with standard aspirin contained one or more lesions and mean lesion score was 23.7 ± 3–5. The severity of ulceration was however reduced by 73 ± 6% in the gastric tissues when aspirin was given micronized and co-grinded with collagen. This was confirmed by evaluating microphotographs of gastric mucosa stained. In particular, we have found that standard aspirin formulation leads to production of severe erosions marked by the presence of heterogeneous mixture of tissues retracting from the mucosal surface. A variety of surface epithelial changes in shape, size, and orientation, accompanied by marked loss of surface mucus epithelial cell were found. Marked disorganization and atrophy of glands were invariably noted. This seems to be prevented when aspirin is micronized and co-grinded with collagen, which maintains the adherent mucus lining resisting the erosion of glandular cells. No significant gastric mucosal lesion was observed in control group of rats.

## Conclusion

Our data show that sublingual formulation of aspirin micronized and co-grinded with collagen displays a better pharmakinetic profile compared with standard crystalline aspirin. This effect was accompanied by attenuated direct gastric ulcerogenic effect of the new formulation and by non-inferiority profile on TXB2 serum and urinary levels after a 7-day treatment compared to the standard formulation of aspirin. The potential remaining warning on gastrointestinal bleeding due to inhibition on prostaglandin-related production of protective gastric mucus of the new formulation is to be better clarified. This represents a new option for better aspirin treatment in the prevention of thromboembolic disorders.

## Supplementary material

Supplementary material is available at *European Heart Journal—Cardiovascular Pharmacotherapy* online.

**Conflict of interest:** none declared.

## Supplementary Material

Supplementary Material
