# Supplementary material for: Aspirin wears smart
Source: Eur Heart J Cardiovasc Pharmacother. 2017 May 15;3(4):185–8. doi: 10.1093/ehjcvp/pvx017 (PMC5843133; doi:10.1093/ehjcvp/pvx017)
Supplement: Supplementary Material [file aspirin_supplementary_material_pvx017.docx]

**SUPPLEMENTARY MATERIAL**

**MATERIALS AND METHODS**

**Materials**

Type I bovine collagen hydrolysate was purchased from LapiGelatin (Pistoia, Italy). Aspirin (acethylsalicilic acid), maltodextrin, microcrystalline cellulose, sodium carboxy-methyl starch, sucralose, magnesium stearate were purchased fron Sigma-Aldrich (Milan, Italy)

**Preparation of aspirin formulations**

A mixture of hydrolyzed bovine collagen and crystalline aspirin was micronized and co-grinded in a ratio of 1 to 1 by means of a pin rotor mortar which leads to micronization of powder particles at <40 μ (Pulverisette 14, Fisher, Germany). Optical microscopy showed, after 20 minutes, the disappearance of micro crystals of aspirin, replaced by amorphous particles surrounded by collagen. The amorphization of aspirin was confirmed by means of Differential Scanning Calorimetry (DSC), performed with Perkin Elmer apparatus DSC7, calibrated with Indium. The samples were examined with a scanning speed of 5.0 C / min. Granular aspirin was tested highlighting the melting peak, in the range 133.9 - 136.8, which characterizes the crystalline building. In contrast, when evaluating aspirin micronized and co-grinded with bovine collagen hydrolysate via DSC, the melting peak between 135 ° C and 138 ° disappeared. The amorphization of aspirin after co-grinding with collagen was also confirmed by Raman MicroSpectroscopy as described in the Figure 1 .

In a second step, standard crystalline aspirin or micronized and co-grinded with collagen powders were used to obtain oral and sublingual formulations (tablets) by means of mechanical compression (Ronchi, Italy) under Good Manufacturing Procedure (GMP- Institute of Research for Food Safety & Health, University of Catanzaro). Microcrystalline cellulose, sodium carboxy-methyl starch, sucralose, magnesium stearate thus were used as eccipients. When required, maltodextrin was used to get the final weight for each tablet homogeneous, as well as in the formulations for placebo.

**Formulation n. 1 - Tablets for oral and sublingual use, each containing 50 mg of crystalline acetylsalicylic acid:**

Aspirin 50 mg

Maltodextrin 50 mg

Microcrystalline cellulose 40 mg

Sodium carboxy-methyl starch 10 mg

Aroma Amarena 6 mg

Sucralose 5 mg

Magnesium stearate 3 mg

**Formulation n. 2 - Tablets for oral or sublingual use, each containing 100 mg of crystalline acetylsalicylic acid:**

Aspirin 100 mg

Maltodextrin 100 mg

Microcrystalline cellulose 40 mg

Sodium carboxy-methyl starch 10 mg

Aroma Amarena 6 mg

Sucralose 5 mg

Magnesium stearate 3 mg

**Formulation n. 3 - Tablets for oral or sublingual use, each containing 50 mg of acetylsalicylic acid micronized and co-grinded with collagen**

Aspirin 50 mg

Collagen 0 degrees Bloom 50 mg

Microcrystalline cellulose 40 mg

Sodium carboxymethyl starch 10 mg

Aroma Amarena 6 mg

Sucralose 5 mg

Magnesium stearate 3 mg

**Formulation n. 4 - Tablets for oral or sublingual use, each containing 50 mg of acetylsalicylic acid micronized and co-grinded with collagen**

Aspirin 100 mg

Collagen 0 degrees Bloom 100 mg

Microcrystalline cellulose 40 mg

Sodium carboxymethyl starch 10 mg

Aroma Amarena 6 mg

Sucralose 5 mg

Magnesium stearate 3 mg

Placebo was obtained by substitution of aspirin and/or collagen in tablets with maltodextrin.

**Dissolution studies**

In vitro dissolution of standard as well as micronized collagen co-grinded aspirin 50 and 100 mg tablets was investigated. The products were tested under the current USP/NF monograph dissolution procedure for aspirin tablets and USP general test. To capture the dissolution profiles of the two products, samples were taken at 1, 3, 6 and 15 min in addition to the USP required with a *Q* point at 30 min. A modified test procedure was also run by using dissolution media prepared (in accordance with the USP reagents section on the preparation of buffers) at pH 1.2 and 6.8 , respectively.

Calibration solutions containing 1 mg/mL of aspirin were prepared in the same pH buffer as the dissolution being performed. Prior to measurement, the isosbestic point for acetylsalicylic acid was determined using 1 mg/mL solutions of each. The samples were collected and filtered through a 10 filter prior to measuring at the predetermined isosbestic point, in a Cary Model 50 spectrophotometer. The calibration was current for the equipment employed for the analyses which were conducted in conformity with GMP requirements.

The aspirin tablet formulations were dissolved to an extent of 92.5 and 99.2 %, respectively, at 15 min, while at the same time the aspirin standard tablet was dissolved to an extent of 47.6 and 82.8%, at the two pH values, respectively. The in vitro dissolution data show that the micronized aspirin tablet enters solution faster than the aspirin standard tablet at pH 1.2 and 6.8. The rate of dissolution for both products were dependent upon the pH of the dissolution medium, with a lower pH producing a slower rate of dissolution.

**Pharmacokinetic studies**

A phase I, randomized, double blind, placebo-controlled study was performed for evaluating the pharmacokinetic of oral aspirin 50 and 100 compared to sublingual 50 and 100 mg either crystalline in standard formulation as well as micronized co-grinded with collagen and placebo, which were administered to 120 healthy subjects. The study complied with the principles of the Good Clinical Practice International Conference on Harmonization rules and was approved by the University Ethics Committee (EudraCT N. 2013-002980-24). Each study participant provided written informed consent.

Healthy volunteers were randomized using computerized random-number generation by an independent investigator on a double-blind basis and randomly assigned to 12 groups (10 subjects each) according to the type and dose of aspirin or placebo (table 1 summarizes subjects demographics). In particular, subjects assigned to groups A, B,C and D received standard aspirin 50 and 100 mg both orally and sublingually, respectively. Groups E, F G and H received 50 and 100 mg of oral or sublingual formulation of aspirin micronized and co-grinded with collagen. Further four groups I, L,M and N received oral administration of placebo. All subjects enrolled received aspirin or placebo after an overnight fast (only water was allowed), for seven consecutive days, at 08.00, with 200 mL of water. On days 1 and 7, the volunteers were admitted to the clinical research center for blood and urine sample collection and the administration of the first and last dose of the drug, which occurred under the supervision of the investigator. They were also instructed how to take aspirin or placebo at their home on days two to six. On day 7, volunteers returned the box for compliance assessment by pill counts. Pill count adherence was 100% and no enrolled subject was excluded from the study. In addition, compliance was monitored by contacting the subjects by telephone at each time of administration at home. On day 1 and day 7, at 08.00, fasted volunteers underwent a venous blood sample collection (baseline, pre-drug) and then received a witnessed administration of aspirin or placebo. No food was allowed for at least 6 h post-dose. Water was allowed as desired except for 1 h before and after drug administration.

On day 1 and day 7, blood sampling was performed before the drug was given and at multiple time-points to assess acetylsalicylic acid and salicylic acid plasma levels by liquid chromatography (LC)/MS/MS and serum TXB_2_ levels (by means of ELISA immunoassay; BioRad, Milan). Before administration of aspirin formulations or placebo and after the sixth dose, all subjects performed a 24-h urine collection in order to study the effect of aspirin on urinary levels of 11-dehydro-TXB_2_ ((by means of ELISA immunoassay; BioRad, Milan).

Plasma acetylsalicylic acid and salicylic acid and serum TXB_2_ and urinary 11-dehydro-TXB_2_ were validated for precision and accuracy according to EMA guidelines. The serum levels of TXB_2_ and urinary 11-dehydro-TXB_2_ in healthy volunteers were 385.30 ± 1125 ng/ml and 365,21 + 138 pg/mg creatinine, respectively. Changes of these parameters were assumed for calculating the % response after aspirin treatment.

**Assessment of acetylsalicylic acid and salicylic acid**

Blood samples were collected using precooled vacutainer tubes containing sodium fluoride/potassium oxalate (Greiner Bio-One, Frickenhausen, Germany) to prevent the hydrolysis of acetylsalicylic acid to salicylic acid; then, plasma samples, combined with deuterated(d4)-acetylsalicylic acid and d4-salicylic acid (Santa Cruz Biotechnology, Dallas, TX, USA),were analyzed by using LC-MS/MS [Waters Alliance 2795 LC coupled to a Micromass 4 Pt triple-quadrupole mass spectrometer (TQuattro-Pt; Waters, Milan, Italy) equipped with a Z-Spray ESI source, operating in negative ion mode]. Analysis was conducted by monitoring the precursor ion to product ion, m/z 136.90 > 92.90 for acetylsalicylic acid and salicylic acid and m/z 140.9 > 96.90 for d4- acetylsalicylic acid and d4-salicylic acid. Acetylsalicylic acid and salicylic acid were separated by reverse-phase HPLC and eluted with retention times of 0.86 and 3.15 min, respectively.

**Table 1S**

**Demographics of healthy volunteers enrolled into the study**

| Group | N. | Age (years) | Gender  (man / woman) | Body weight  (Kg) | Body Mass Index (Kg/m^2^) | Smoking | Concomitant treatment |
| --- | --- | --- | --- | --- | --- | --- | --- |
| A – crystalline aspirin oral 50 mg | 10 | 32 + 6 | 5 M and 5 W | 68 + 8 | 23 + 2 | 0 | 0 |
| B – crystalline aspirin oral 100 mg | 10 | 34 + 4 | 5 M and 5 W | 66 + 6 | 24 + 4 | 0 | 0 |
| C – crystalline aspirin sublingual 50 mg | 10 | 34 + 5 | 5 M and 5 W | 65 + 7 | 22 + 3 | 0 | 0 |
| D – crystalline aspirin sublingual 50 mg | 10 | 31 + 6 | 5 M and 5 W | 65 + 6 | 22 + 4 | 0 | 0 |
| E – micronized aspirin oral 50 mg | 10 | 35 + 5 | 5 M and 5 W | 68 + 6 | 25 + 4 | 0 | 0 |
| F – micronized aspirin oral 100 mg | 10 | 34 + 5 | 5 M and 5 W | 65 + 8 | 23 + 3 | 0 | 0 |
| G – micronized aspirin sublingual 50 mg | 10 | 35 + 5 | 5 M and 5 W | 68 + 8 | 24 + 3 | 0 | 0 |
| H – micronized aspirin sublingual 100 mg | 10 | 33 + 6 | 5 M and 5 W | 66 + 8 | 24 + 5 | 0 | 0 |
| A – crystalline placebo oral 100 mg | 10 | 33 + 5 | 5 M and 5 W | 66 + 6 | 23 + 3 | 0 | 0 |
| A – crystalline placebo sublingual 100 mg | 10 | 35 + 5 | 5 M and 5 W | 68 + 7 | 23 + 3 | 0 | 0 |
| A – micronized placebo oral 100 mg | 10 | 32 + 4 | 5 M and 5 W | 67 + 7 | 25 + 3 | 0 | 0 |
| A – micronized placebo sublingual 100 mg | 10 | 34 + 5 | 5 M and 5 W | 66 + 8 | 22 + 4 | 0 | 0 |

**Detection of Ulcer Index Score in rats**

Sprague–Dawley male rats (140–180 g) were used for this study. The animals were housed under standard conditions of temperature of 25 ± 2 °C, relative humidity 45–55% and 12 h light/dark cycle, and fed with standard rodent feed (Charles River, Milano, Italy) and water. Food was withdrawn 18–24 h before the experiment though water was allowed *ad libitum* and allocated to different experimental groups, each of six rats. All animal experiments were conducted with the permission from Institutional Animal Ethics Committee of University of Catanzaro, Italy.

Crystalline standard aspirin or aspirin micronized and co-grinded with collagen in a dose of 400 mg/kg (20 mg/ml) were administered to the animals on the day of the experiment and ulcers were scored after 5 h. The animals were sacrificed and the stomach was then excised and cut along the greater curvature, washed carefully with 5.0 ml of 0.9% NaCl, and ulcers were scored by a person unaware of the experimental protocol in the glandular portion of the stomach. Ulcer index (UI) was calculated by adding the total number of ulcers and the total severity of ulcers per stomach. The pooled group ulcer score was then calculated according to the method of Taylor (1982; see Ref. 11). The comparative response between standard crystalline and micronized co-grinded aspirin on experimental ulcers and the protection % was calculated using the following formula:


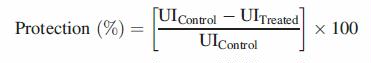


**Histological studies in rats undergoing acute aspirin treatment**

Samples from the gastric mucosa of each stomach were fixed in 10% formalin solution and embedded in paraffin after treatment with 400 mg/Kg of crystalline standard aspirin as well as in rats treated with aspirin micronized and co-grinded with collagen. Serial sections of 5-μm thickness were obtained and stained with hematoxylin/eosin (HE) to evaluate gastric morphology.

**Statistical analysis**

The data were expressed as mean + SD, unless otherwise stated, and statistical comparisons were made by parametric tests (Student’s t-test or repeated-measures analysis of variance followed by the Student-Newman-Keuls test). A probability value of P < 0.05 was considered to be statistically significant.
